# Evolution in functionality of a metastatic pancreatic neuroendocrine tumour (pNET) causing Cushing’s syndrome: treatment response with chemotherapy

**DOI:** 10.1186/1472-6823-14-70

**Published:** 2014-08-24

**Authors:** Surya Panicker Rajeev, Steffan McDougall, Monica Terlizzo, Daniel Palmer, Christina Daousi, Daniel J Cuthbertson

**Affiliations:** 1 Department of Obesity and Endocrinology, University Hospital Aintree, Liverpool L9 7AL, UK; 2 Department of Pathology, University Hospital Aintree, Liverpool L9 7AL, UK; 3 Department of Molecular and Clinical Cancer Medicine, Institute of Translational Medicine, University of Liverpool, Liverpool L69 3BX, UK; 4 Department of Obesity and Endocrinology, Institute of Ageing and Chronic Disease, University of Liverpool, Liverpool L69 3BX, UK

**Keywords:** Pancreatic neuroendocrine tumour, Cushing’s syndrome, Chemotherapy

## Abstract

**Background:**

We report the case of a patient who had a non-functional metastatic pancreatic neuroendocrine tumour (pNET), which changed in functionality during the course of the disease. This case demonstrates the effectiveness of conventional cytotoxic chemotherapy in the management of select group of patients with this rare, challenging condition.

**Case presentation:**

Our patient was a 34 year old man under oncology follow up, diagnosed with a non-functional metastatic pancreatic neuroendocrine tumour treated with a Whipple’s procedure two years ago. Despite treatment with somatostatin analogues and sunitinib, a tyrosine kinase inhibitor, he had demonstrated radiological progression of his metastatic disease. He now presented with a short history of Cushing’s syndrome. A presumptive diagnosis of a rapidly progressive, metastatic, functional pNET with ectopic ACTH production was made, confirmed biochemically and with liver biopsy. The proliferative index, Ki-67 of 20% of the liver biopsy prompted us to treat him with conventional cytotoxic chemotherapy using streptozocin, 5-fluorouracil and doxorubicin. Prior to its administration clinical and biochemical control of the hypercortisolemic state was achieved with metyrapone. However the clinical, biochemical and radiological response to chemotherapy was so dramatic obviating the need for metyrapone therapy.

**Conclusions:**

Non-functional pNETs may evolve in their clinical and biologic behaviour producing functional hormonal syndromes. Chemotherapy may be an effective therapeutic modality in such circumstances.

## Background

ACTH secretion in functional pNETS is rare and it is even more rare for non-functional pNETs to evolve into ACTH secreting functional tumours with only very few cases reported in literature. We describe such an interesting and rare case of evolution in functionality of a non-functional pNET. Apart from control of hypercortisolaemic state, preventing disease progression can be a real challenge despite various therapeutic modalities being increasingly used in the last decade. We report the case of a young patient who had a non-functional pNET, which later started secreting ACTH, highlighting the importance of being very vigilant during the follow up of such patients. Despite novel chemotherapeutic options and PRRT (Peptide Receptor Radionuclide Therapy), conventional chemotherapeutic agents still have a very important role and should be tried if the above agents fail to control disease state as demonstrated by our case.

## Case presentation

We report the case of a 34-year-old man who presented with a one-month history of lethargy, generalised upper and lower limb weakness and significant weight gain. He had been recently diagnosed with type 2 diabetes mellitus and initiated on pre-mixed insulin injections 30 units twice daily (HbA1c 85 mmol/mol).

He had been diagnosed with a pancreatic neuroendocrine tumour (pNET) two years previously, having presented with jaundice and abdominal pain. An abdominal CT had demonstrated tumour in the head of the pancreas with loco-regional metastases (peripancreatic lymph nodes and nine hepatic metastatic lesions varying in size from 7–18 mm) for which he underwent a Whipple’s procedure with resection of lymph nodes and an intra-operative liver biopsy. Measurement of a full fasting gut hormone profile showed elevated chromogranin A but was otherwise normal, consistent with a non-functional tumour. Immunohistochemistry of the pancreatic specimen was positive for chromogranin and synaptophysin with a Ki-67 index of 2% confirming the diagnosis of a Grade 2 pNET (ACTH staining not performed); the liver biopsy appearances were similar morphologically. A careful family history of endocrine tumours or endocrine disorders had been unremarkable.

On follow up imaging he had developed further liver metastases, so was commenced on a long-acting somatostatin analogue (Lanreotide), for its anti-proliferative potential, and a tyrosine kinase inhibitor (Sunitinib) was thereafter added upon evidence of further radiological progression.

**Figure 1:**
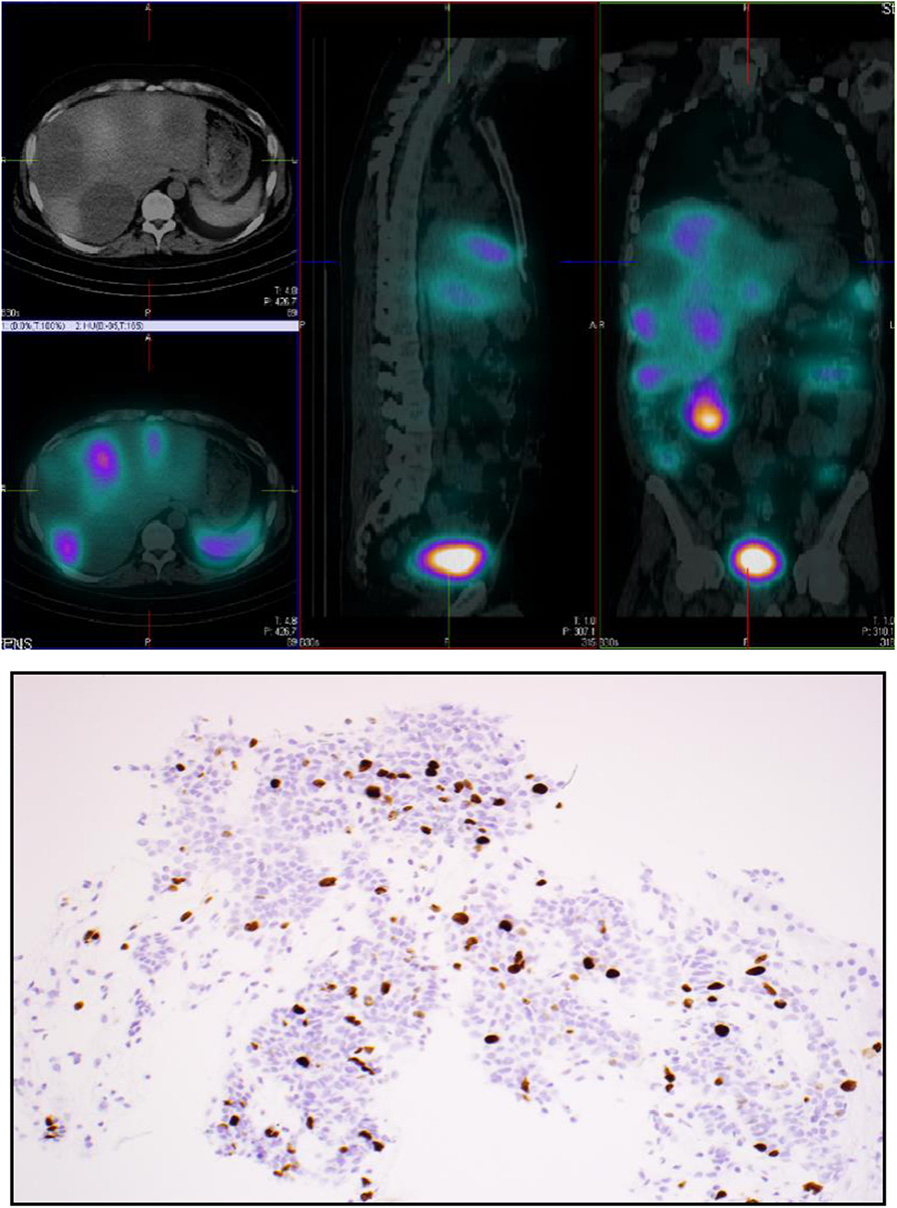
**(upper figure) **^**111**^**Indium-labeled Octreotide scan.** Figure 1 (lower figure) Ki-67 immunostaining of liver metastasis (at recurrence) showing a high proliferative rate of 20%.

Two years subsequently, he presented with cushingoid features with a moon face, easy bruising, abdominal striae, centripetal fat distribution and marked proximal myopathy. Blood pressure was normal. Biochemical investigations revealed serum potassium concentration of 2.5 mmol/l, glucose 17 mmol/l and significantly elevated random serum cortisol of 2003 nmol/L and serum ACTH concentration 50 pmol/L (normal range 2–11 pmol/L). Basal pituitary biochemistry and gadolinium enhanced MRI imaging was otherwise unremarkable. MRI scan of the liver revealed solid and cystic metastatic deposits ranging between 7.8-9.2 cm in segments 6, 7 and 8 indicating further progression despite sunitinib.^111^In-labeled octreotide scanning demonstrated somatostatin receptor positive disease in five of his liver metastatic deposits but not in any other sites (Figure 1).

**Figure 2:**
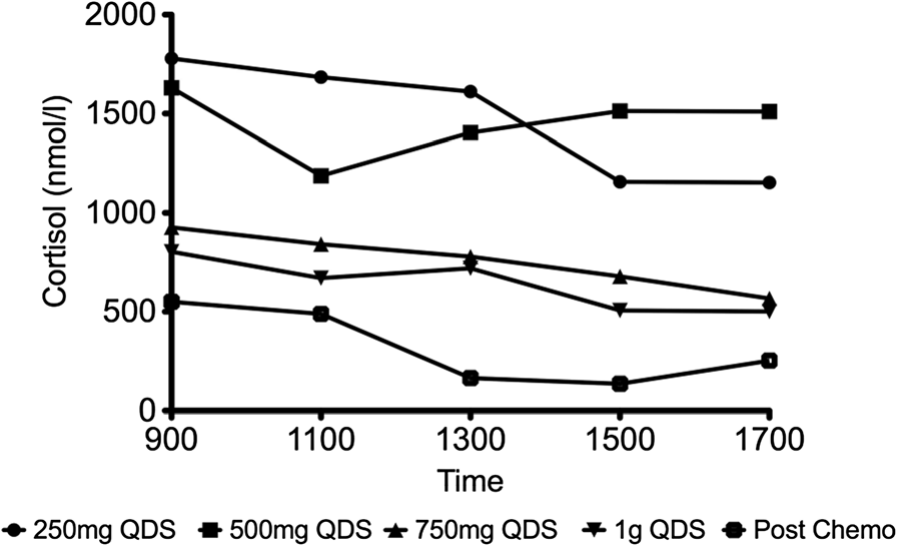
Cortisol day curves demonstrating cortisol concentrations on incremental doses of metyrapone, up to 1 g qds prior to chemotherapy, and effects of chemotherapy with patient having discontinued metyrapone.

A diagnosis of a rapidly progressive, functional, metastatic pNET with ectopic ACTH production causing Cushing’s syndrome was made, with an assumption that the tumour had evolved in its functionality from its previous non-functional status. Treatment options discussed at the supra-regional multidisciplinary team meeting (ENETS Centre of Excellence) considered metyrapone, bilateral adrenalectomy, peptide receptor radionuclide therapy (PRRT) or cytotoxic chemotherapy. Mutational analysis of the gene for multiple endocrine neoplasia type 1 (MEN 1) was negative.A repeat liver biopsy was performed to provide an accurate histological grade of the liver metastases, on the premise that primary NETs, and their synchronous/metachronous metastases, frequently differ in grade and proliferative index (Ki-67). Immunohistochemistry was strongly positive for chromogranin and synaptophysin with a Ki-67 index of 20% (ACTH staining not performed) (Figure 1). The treatment decision based on this result was to administer conventional cytotoxic chemotherapy with streptozocin, 5-fluorouracil and doxorubicin, with metyrapone given pre-chemotherapy to control the hypercortisolaemic state. Incremental doses of metyrapone (up to 1 g qds) effected a dramatic clinical response (with resolution of his symptoms and reduction of his insulin dosage) and an equally dramatic biochemical response (normalisation of serum potassium and lowering of mean cortisol concentrations on cortisol day curves) prior to his chemotherapy.Three months after starting his chemotherapy, he had managed to completely discontinue his metyrapone with excellent mean cortisol concentrations on his cortisol day curve of 315 nmol/L (Figure 2 graph), with serum ACTH concentration of 11pmol/l. He discontinued all insulin injections with excellent glycaemic control (HbA1c 53 mmol/mol) and cross-sectional imaging (abdominal CT) showing a dramatic reduction in the size of the hepatic metastases with more necrotic/cystic contents than previously. He remains clinically stable three months later.

## Discussion

pNETs are rare with a reported incidence of 1-2/100,000. They may be functional tumours, with an associated clinical syndrome, or non-functional, which despite not producing any hormone-specific symptoms, frequently either secrete other peptides (such as pancreatic polypeptide, chromogranin A, neuron specific enolase) or may stain positively on immunohistochemistry for certain hormones.

The most common functional pancreatic NET syndromes relate to insulinoma and gastrinoma. ACTH secretion occurs only rarely, presenting with features of Cushing’s syndrome, but more than 95% are malignant with an average age at diagnosis of 50–55 years and an equal sex distribution [1]. Functional pNETs account for 4-16% of patients with ectopic ACTH secretion [1]. The diagnostic approach is similar to that in suspected Cushing’s syndrome, first to confirm excess cortisol production and then to localise the tumour source.

Even more rarely, initially non-functional pNETs have been reported to evolve into functional tumours. In a series of 353 patients described by Wynick *et al.*, 24 patients demonstrated *de novo* evidence of secretion of a second hormone during the course of the disease (median delay of 19 months and 92% reflecting liver metastases) [2]. Several reports have highlighted clinical cases of ectopic ACTH/Cushing’s syndrome, where secondary deposits stained strongly positive for ACTH despite a primary tumour being negative on ACTH staining [3]. In our case, ACTH staining was not deemed necessary with biochemical confirmation, considering the high cost involved and availability of ACTH staining in only a single United Kingdom centre. However, as above, our patient presented after 24 months of the initial diagnosis of non-functional pNET with hepatic metastatic disease and *de novo* Cushing’s syndrome, highlighting evolution in tumour functionality should be considered during follow up of patients with pNETs.

Treatment aims are twofold involving biochemical control of hypercortisolaemia and preventing disease progression. Adrenal blocking agents like metyrapone, or bilateral adrenalectomy in patients who fail to respond to metyrapone therapy, may be used to address hypercortisolaemia. Medical treatment of the neuroendocrine tumour may involve long acting somatostatin analogues (SSA) or occasionally alpha interferon may be effective. Surgery is recommended even in the presence of metastatic disease, including localized liver metastases, if potentially resectable, and symptomatic control of the hormone excess state through cytoreductive surgery or radiofrequency ablation. However, our patient had large, multiple liver metastatic deposits and hence surgery was not deemed to be an option.

In patients with advanced, surgically non-resectable, progressive pNETs, everolimus [4] and sunitinib [5] are novel chemotherapeutic options and PRRT (Peptide Receptor Radionuclide Therapy) may also have a role. Ki-67 index is important in deciding treatment options and triage patients into the most appropriate treatment arm. Conventional chemotherapy with streptozocin, 5- fluorouarcil and doxorubicin still has a place as in the management of some patients with more aggressive tumours. Our patient failed to respond to SSAs and sunitinib, but had an excellent treatment response to conventional chemotherapy.

## Conclusions

This clinical case offers several important learning points. Firstly, there should be vigilance to the possibility of an evolution or change of functionality of pNETs, during tumour progression, particularly in non-functional pNETs considering their pluripotency with awareness of the various clinical syndromes that may arise. Secondly, repeat histological examination of metastatic deposits may indicate different biological behaviour of the tumour and this may modify the treatment regime. In the case described, we observed the evolution towards Cushing’s syndrome from a non-functional tumour, with repeat histological findings modifying our treatment approach and facilitating effective treatment with chemotherapy. The different proliferative potential may represent inherent heterogeneity within the tumour or across different anatomical sites but may reflect evolution in the histology towards a higher grade.

## Consent

Written informed consent was obtained from the patient for publication of this Case report and any accompanying images. A copy of the written consent is available for review by the Editor of this journal.

## Abbreviations

pNET: Pancreatic neuroendocrine tumour; PRRT: Peptide receptor radionuclide therapy; MEN: Multiple endocrine neoplasia; SSA: Somatostatin analogues; ACTH: Adrenocorticotrophic hormone.

## Competing interests

The authors declare that they have no competing interest.

## Authors’ contributions

SPR drafted the initial manuscript, which was revised by DJC and CD. SM was involved in the management of our patient and helped in preparing the manuscript. MT performed the immunohistochemistry. DHP was involved in the oncological management and helped in the revision of the script. All authors have read and approved the final manuscript.

## Pre-publication history

The pre-publication history for this paper can be accessed here:

http://www.biomedcentral.com/1472-6823/14/70/prepub
